# Detection of β-Lactamase Resistance and Biofilm Genes in *Pseudomonas* Species Isolated from Chickens

**DOI:** 10.3390/microorganisms10101975

**Published:** 2022-10-06

**Authors:** Hams M. A. Mohamed, Sulaiman Mohammed Alnasser, Hanan H. Abd-Elhafeez, Meshal Alotaibi, Gaber El-Saber Batiha, Waleed Younis

**Affiliations:** 1 Department of Microbiology, Faculty of Veterinary Medicine, South Valley University, Qena 83523, Egypt; 2 Department of Pharmacology and Toxicology, Unaizah College of Pharmacy, Qassim University, Buraydah 52571, Saudi Arabia; 3 Department of Cells and Tissues, Faculty of Veterinary Medicine, Assiut University, Assiut 71526, Egypt; 4 Department of Pharmacy Practice, College of Pharmacy, University of Hafr Albatin, Hafr Albatin 39524, Saudi Arabia; 5 Department of Pharmacology and Therapeutics, Faculty of Veterinary Medicine, Damanhur University, Damanhur 22511, Egypt

**Keywords:** *Pseudomonas* species, antimicrobial resistance, biofilm formation, clove oil, plasmid, PCR, *AmpC* β-Lactamases

## Abstract

Bacteria of the genus *Pseudomonas* are pathogens in both humans and animals. The most prevalent nosocomial pathogen is *P. aeruginosa*, particularly strains with elevated antibiotic resistance. In this study, a total of eighteen previously identified *Pseudomonas* species strains, were isolated from chicken. These strains were screened for biofilm formation and antibiotic resistance. In addition, we evaluated clove oil’s effectiveness against *Pseudomonas* isolates as an antibiofilm agent. The results showed that *Pseudomonas* species isolates were resistant to most antibiotics tested, particularly those from the β-lactamase family. A significant correlation (*p* < 0.05) between the development of multidrug-resistant isolates and biofilms is too informal. After amplifying the *AmpC*-plasmid-mediated genes (*bla_CMY_*, *bla_MIR_*, *DHA*, and *FOX*) and biofilm-related genes (*psld*, *rhlA*, and *pelA*) in most of our isolates, PCR confirmed this relationship. Clove oil has a potent antibiofilm effect against *Pseudomonas* isolates, and may provide a treatment for bacteria that form biofilms and are resistant to antimicrobials.

## 1. Introduction

Gram-negative bacteria belonging to the *Pseudomonas* genus, including *Pseudomonas aeruginosa*, are frequently found in the environment and isolated from various chronic infections in both humans and animals [1]. The bacterium can inhabit soil and infect animals and plants. In addition, *Pseudomonas aeruginosa (P. aeruginosa*) is considered an opportunistic organism in avian species. Under normal environmental conditions; however, under various stressors, this organism becomes pathogenic. Avian *P. aeruginosa* infections are associated with respiratory symptoms, diarrhea, and mortality with resulting severe losses in the poultry industry [2].

Several studies have demonstrated *Pseudomonas* infection in humans through occupational contact with poultry or related products. [3,4]. *P. aeruginosa* is a significant pathogens in human infections [5]. In addition, *P. aeruginosa* is one of the most common causes of acute nosocomial infections, especially in patients admitted to the intensive care unit. The most prevalent and dangerous cause of chronic respiratory infections or other chronic underlying diseases is *P. aeruginosa* [6].

Another *Pseudomonas* species, *P. fluorescens* is a naturally occurring bacteria in the mouth, stomach, and lungs, among other areas [7]. There is an eccentric link between *P. fluorescens* and human diseases, especially bacteremia. Numerous articles have discussed the importance of *P. aeruginosa* and *P. fluorescens* nosocomial infections [7,8].

These bacteria continue to develop new defense mechanisms against various antibiotics and cause them to become multidrug resistant. The emergence of antimicrobial resistant bacteria poses a serious threat to public health and has led to increased hospital morbidity and mortality [9].

One of the most important resistance mechanisms was the plasmid-mediated synthesis of enzymes that render antibiotics inactive by hydrolyzing their β-lactam rings, which increases the number of point-mutations and leading to conventional broad-spectrum antibiotics resistance [10]. We refer to these resistances as extended spectrum resistances. Lower, third, and fourth generation cephalosporins, trimethoprim–sulfamethoxazole, aminoglycosides, and fluoroquinolones are all hydrolyzed by extended-spectrum β-lactamases (ESBL) [11,12].

β-lactam resistance associated with multiple mechanisms includes mutational changes in the PBP3 protein, decreased antibiotic uptake, increased export, and degradation of the antibiotic molecule [13]. In addition, horizontal gene transfer may inherit antibiotic-degrading enzymes (β-lactamases) from other bacteria [14]. Biofilm production may play a role in resistance as well [15], these resistance are mediated by AmpC genes (*bla_FOX_, bla_DHA_, bla_CMY_,* and *bla_ACC_*) which are prospective sources for the extensive dissemination of antibiotic resistance [16].

Biofilm, is considered one of several important mechanisms for antibiotic resistance [17]. Alginates and Pel are two of the three exopolysaccharides in the *P. aeruginosa* biofilm matrix. Pel, which is made up of a repeated pentasaccharide, is the most important and is the first stage in the formation of biofilms and the preservation of their structural integrity [13]. The next stage of biofilm spread is maturation, for which the primary gene responsible is the second quorum-sensing regulon (*rhlA*) [18].

Concerns about the connection between biofilm production and antibiotic resistance have been raised by biomedical researchers [19,20]. In the fight against biofilms, natural antimicrobials such as essential oils (EOs) have emerged as an effective substitute for synthetic antibiotics [21]. EOs are complex liquid mixtures of volatile chemicals that are distilled from various plant parts, known to contain antibacterial compounds [22,23].

Clove essential oil drived from the dried floral bud of the plant *Syzygium aromaticum* of the family Myrtaceae, which exhibits antimicrobial a and antioxidant properties [24,25]. Additionally, clove oil has a potent anti-biofilm effect against *Pseudomonas* isolates, and may provide a treatment for MDR and biofilm-forming bacteria [26,27,28].

The current study aimed to assess the susceptibility of *Pseudomonas* strains to various antimicrobials, how the capacity of *Pseudomonas* to form a biofilm was affected, and the detection of β-lactamase resistant genes by PCR to assess the antibiofilm activity of clove oil against strains of *Pseudomonas* that form biofilms.

## 2. Materials and Methods

### 2.1. Consent for Participation

Samples were collected from owners after asking for their permission to collect samples from their homes in small communities, keep them, and use them in this study. Owners’ consent was obtained for participation, and a sample of consent was attached.

### 2.2. Ethical Approval

The research was conducted in accordance with all relevant norms and laws. Both the South Valley University National Ethics Committee and the veterinary authorities in Egypt’s South Valley Province provided their approval. The Use and Care of Experimental Animals in the Faculty of Veterinary Medicine, South Valley University were approved by Egypt’s Committee. All procedures were carried out in conformity with all applicable rules and regulations. No. 45/08.09.2022.

### 2.3. ARRIVE Principles

The research was completed in accordance with the ARRIVE (Animals in Research: Reporting In Vivo Experiments) standards [29].

### 2.4. Bacterial Strains

The eighteen *Pseudomonas* strains used in this study were discovered and previously identified by Shahat et al. [30]. Briefly, during outbreaks of respiratory infection in chickens, strains were isolated from poultry farms and identified serologically according to Glupczynski et al. [31], based on the manufacturer’s recommended protocol (Bio-Rad, France). This collection of bacteria contained eleven isolates of *P. fluorescens* and seven isolates of *P. aeruginosa.*

According to Legakis et al. [32], *P. aeruginosa* is divided into groups based on *P. aeruginosa* O antisera and the international antigen typing scheme (IATS). In this study 1 isolate was *P. aeruginosa* O2, 2 isolates were *P. aeruginosa* O6, 3 isolates were *P. aeruginosa* O11, 1 was *P. aeruginosa* O10 and 11 isolates were *P. fluorescens*. These isolates were confirmed by PCR amplification of a housekeeping gene (*16S rDNA* gene).

### 2.5. Antimicrobial Susceptibility Testing

According to John et al. [33], the zones of inhibition in the bacterial isolates were assessed using the disc diffusion test. Various antimicrobial classes were tested including **aminoglycosides** (gentamicin 10 µg and amikacin 30 µg), **penicillin** (piperacillin 75 µg), **tetracycline** (tetracycline 30 µg), **quinolones** (ciprofloxacin 5 µg, levofloxacin 5 µg, and norfloxacin 10 µg), **sulfonamides** (trimethoprim–sulfamethoxazole 25 µg), **cephalosporins** (cefotaxime 30 µg and cefazoline 30 µg), **carbapenem** (meropenem and imipenem (10 µg)) and **chloramphenicol** (chloramphenicol 30 µg). The results were interpreted in accordance with the Clinical and Laboratory Standards Institute (CLSI) [34]. and European Committee on Antimicrobial Susceptibility Testing (EUCAST) [35] standards (susceptibility/intermediate/resistance). Multidrug-resistant (MDR) strains were defined by their resistance to three or more antimicrobial classes.

### 2.6. Phenotypic and Genotypic Screening for AmpC β-Lactamases

#### 2.6.1. Screening for AmpC β-lactamases

According to CLSI guidelines, the isolates were initially examined for the potential production of AmpC β-lactamases [34]. Cefotaxime (30 μg) was tested for antibiotic susceptibility, and organisms that were resistant to it (showing a zone of inhibition with a diameter of ≤18 mm) were screened as potential producers of AmpC β-lactamase.

#### 2.6.2. Confirmation of AmpC β-lactamase Production

##### Boronic Acid Test

An inhibitor-based confirmatory test was used during screening to identify positive AmpC β-lactamases producers (boronic acid test). On a Muller Hinton agar plate, a culture of the tested organism was created using a 0.5 McFarland bacterial growth standard. Two 30 μg cefotaxime discs were incubated on a plate of Muller Hinton agar culture, with one of them receiving phenyl boronic acid treatment (400 g). The chosen antibiotic disc (cefotaxime) was evaluated in combination with phenyl boronic acid; when compared to the test without the combination (antibiotic disc alone), the inhibition zone increased by 5 mm, and the isolate was recognized as having a positive boronic acid test [36].

##### Disk Test

*E. coli* ATCC 25,922 was obtained as the control strain from the faculty of agriculture at Ain Shams University in Giza, Egypt.

The Muller Hinton agar plate (HiMedia, Mumbai, India) containing the *E. coli* ATCC 25,922 culture was used in the disc approximation test. A saline solution was used to moisten the sterile disc before the tested bacteria colonies were added. The cefotaxime disc was placed next to the inoculated discs., The plates were inverted and incubated aerobically at 37 °C for 24 h. Any flattening or indentation of the zone of inhibition (ZOI) indicated the production of AmpC-lactamase by the isolates [36].

#### 2.6.3. PCR Technique for the Detection of Antimicrobial Resistance Genes

##### Extraction of Plasmid-Mediated AmpC β-lactamases

The Gene JET plasmid Miniprep kit was used to extract the plasmid from the strains that tested positive in the AmpC enzyme primary screening test (Thermo Fisher Scientific, Waltham, MA, USA, K.0502) The manufacturer’s instructions were followed.

##### Multiplex PCR Testing for the *bla_CMY_* and *bla_MIR_*

The multiplex PCR reaction was conducted in a 25 μL enclosed, with 6.5 μL of PCR water, 12.5 μL of the master mix (Taq PCR (2×) from Thermo Fisher Scientific), 1 μL of each *bla_CMY_* and *bla_MIR_* primer (Table 1), and 2 µL of DNA supernatant. The material was amplified via PCR in the applied Biosystem 2720 thermal cycler under the following conditions: initial denaturation for 2 min at 95 °C, denaturation for 30 s at 95 °C, annealing for 30 s at 60 °C, extension for 30 s at 72 °C, and final extension for 4 min at 72 °C. Then, as previously described by Pfeifer et al. [37], 5 μL of each PCR product was examined using 1.5% (*w*/*v*) agarose gel electrophoresis.

##### Uniplex PCR Testing for DHA and FOX

Uniplex PCR protocol was carried out according Wassef et al. [41]. A 25 µL reaction containing 12.5 µL of Emerald Amp Max PCR Master Mix (Takara, Tokyo, Japan), 1 µL of each primer (20 pmol), 4.5 µL of water, and 6 µL of DNA template was carried out using uniplex PCR primers. An Applied Biosystems 2720 Thermal Cycler was used for the reaction. In 1× TBE buffer at room temperature, the following amplification program was used: initial denaturation at 95 °C for 5 min, 35 cycles of denaturation at 95 °C for 30 s, and annealing at 55 °C for 30 s, the final extension occurred at 72 °C for 10 min. The PCR products were separated using 5 V/cm gradients on 1.5% agarose gel (AppliChem, Darmstadt, Germany, GmbH). For gel analysis, 20 µL of PCR product was placed in each gel slot. Using a gene ruler 100 bp DNA ladder (Fermentas, Thermo, Leipzig, Germany), the fragment sizes were calculated. The gel was photographed using a gel documentation system (Alpha Innotech, Biometra, Hercules, CA, USA), and the data were then analyzed using computer software (GelCompar version) [41].

#### 2.6.4. Biofilm Detection Techniques

##### Microtiter Plate Technique

According Stepanović et al. [42], biofilm growth was inconsistent when using a microtiter plate technique. Here, each strain was inoculated into brain-heart infusion broth (BHI) (Oxoid, Waltham, CA, USA) and allowed to grow for 24 h at 37 °C. After being standardized to an OD600 1 ± 0.05 and diluted 1:100 with sterile (BHI plus 1% glucose (Oxoid), the bacteria were placed in a 96-well polystyrene microplate containing 200 µL of BHI medium. The bacteria were then cultured overnight in this environment to produce a final suspension of 1.5 × 10^7^ CFU/mL. Negative control wells were created using only medium.

##### *P. aeruginosa* ATCC 27853 Positive Reference Strain for Biofilm Formation

After incubation, the plates were quickly inverted and moved to remove the growth medium and most of the bacteria. A pipette was used to place 200 μL of PBS 1× into the wells, which was then removed for pipette-based washing by tilting the plate. This technique was performed two more times. The excess liquid that had accumulated due to condensation was then removed for pipetting by tapping the microplates on absorbent paper.

Biofilm production was evaluated using crystal violet staining (0.5%), performed by adding 150 μL of dye to each well and leaving them at room temperature for 5 min. Using a pipette, extra dye was rinsed away. The remaining dye was solubilized with 200 μL of glacial acetic acid (33% v/v) in each well after the plates had air dried.

Utilizing the ELISA auto reader, (Thermo Fisher, Multiskan™ FC,), staining (OD620) was measured [41]. Finally, biofilm formation was categorized using the following criteria: biofilm was not formed if OD ≤ ODc (negative), was weak if ODc < OD < 2 ×ODc and moderate if 2 × ODc < OD < 4 ×ODc. A biofilm was considered strong at 4 × ODc < OD.

#### 2.6.5. PCR Technique for Biofilm Formation Gene Detection

##### DNA Extraction

The DNA of *Pseudomonas* isolates was extracted according to the directions on the QIAamp DNA mini kit.

##### Uniplex PCR Reaction

A uniplex PCR technique was used to detect the biofilm genes *pslD*, *pelA*, and *rhlA* according to Tawakol et al. [43]. The total volume used for the PCR assays was 25 µL. Amplification used 2 µL of the DNA template, 12.5 µL of PCR master mix, 1 µL of each primer, and 8.5 µL of PCR water. The following amplification protocol was used: initial denaturation at 95 °C for 5 min, 40 cycles of denaturation at 95 °C for 30 s, and temperature-adjusted annealing for *pslD*, *rhlA*, and *pslA* at 58 °C for 30 s, 55 °C for 25 s and 60 °C for 40 s, respectively. The final extension occurred at 72 °C for 10 min.

#### 2.6.6. Essential Oil Chromatography

##### Clove Oil

In this study, a crude clove EO of *S. aromaticum*, was used (Al-Ahram Company, Egypt) which contained no synthetic ingredients and was 100% natural. Dimethyl sulfoxide (DMSO) stock solutions of clove essential oil (EOC) were filtered through 0.45 µm millipore filters (Nalgene, UK) to sterilize the oil, which was then kept at 4 °C until it was needed.

##### Gas Chromatography–Mass Spectrometry (GC-MS) Analysis of Clove Oil

A direct capillary column TG-5MS (30 m × 0.25 mm × 0.25 m film thickness) and a GC-TSQ mass spectrometer (Thermo Scientific, Austin, TX, USA) were used to determine the chemical composition of clove oil. The temperature of the column oven was initially maintained at 60 °C, then increased at a rate of 5 °C/min to 250 °C, maintained for 1 min, and then increased at a rate of 30 °C/min to 300 °C. Helium was used as the carrier gas at a constant flow rate of 1 mL/min while the injector temperature was maintained at 270 °C. An AS3000 autosampler and a split mode GC were used to automatically inject 1 µL diluted samples with a 4-min solvent delay. A range of 70 eV ionization voltages were used to acquire EI mass spectra.

#### 2.6.7. Screening Antibiofilm Effects of Clove Oil on *Pseudomonas* spp.

We determined the minimum inhibitory concentration (MIC) in accordance with Kerekes et al. [44]. To find the MIC values, clove oil was diluted in TSB liquid culture medium (Merck, Darmstadt, Germany) with DMSO. The concentrations of clove oil were 6.25%, 12.5%, 25%, and 50%, We placed 100 μL of each bacterium’s 24 h-old cell suspension diluted with liquid culture medium (10^8^ CFU/mL) into each well of a 96-well microtiter plate, then added 100 µL of diluted clove oil. Clove oil was used in sterile medium for positive controls, while clove oil was used in inoculated growth medium for negative controls. After 24 h of incubation at 30 °C, absorbance was measured at 600 nm. The MIC value was established as an absorbance of less than 10% of the samples from the positive controls, signifying growth suppression of at least 90%. Three replicates were made for each dilution.

##### Antibiofilm Activity of Clove Oil

To assess biofilm formation, the technique used by Peeters et al. [45] was applied. Briefly, 200 µL of 24 h-old bacterial culture with about 10^8^ CFU/mL of cells was injected into polystyrene microtiter plates. After 4 h of cell adhesion at the proper temperatures, the supernatant was removed, and the plates were washed with physiological saline. To prevent complete growth inhibition, 200 μL of fresh medium containing the EO was introduced at various doses.

The plates were then incubated for an additional 24 h to promote the development of a biofilm. Positive controls had inoculated in growth medium but no EOs or their components, whereas negative controls had EOs or their components in the growth medium. The experiment was run a minimum of two times. The inhibition of biofilm formation was evaluated with crystal violet staining. After 24 h of incubation, the supernatant was removed, and the wells were rinsed with physiological saline. The biofilms were fixed with methanol before the supernatant was once more removed. Following the addition of a 0.5% crystal violet (CV) solution to each well, the excess dye was washed off the plates by running them under running water for 20 min. The remaining dye was solubilized with 200 μL of glacial acetic acid (33% *v*/*v*) in each well after the plates had air dried, and the bound CV was released. The absorbance was measured at 590 nm according to the equation below and for each EOC concentration, the biofilm inhibition rate of the initial cell adhesion tests was calculated.

Inhibition rate = (ODgc − ODexp/ODg) × 100(1)

In the above equation, ODexp is the optical density of the cultures incubated with the EOC, and ODgc is the optical density of the growth control culture.

### 2.7. Statistical Analysis

Data processing with SPSS (Version28) was used to compare the values. One-way ANOVA was used for analysis, then the Scheffe and Duncan tests were run with a *p* value < 0.05 indicating a significant difference.

## 3. Results

**Figure 1:**
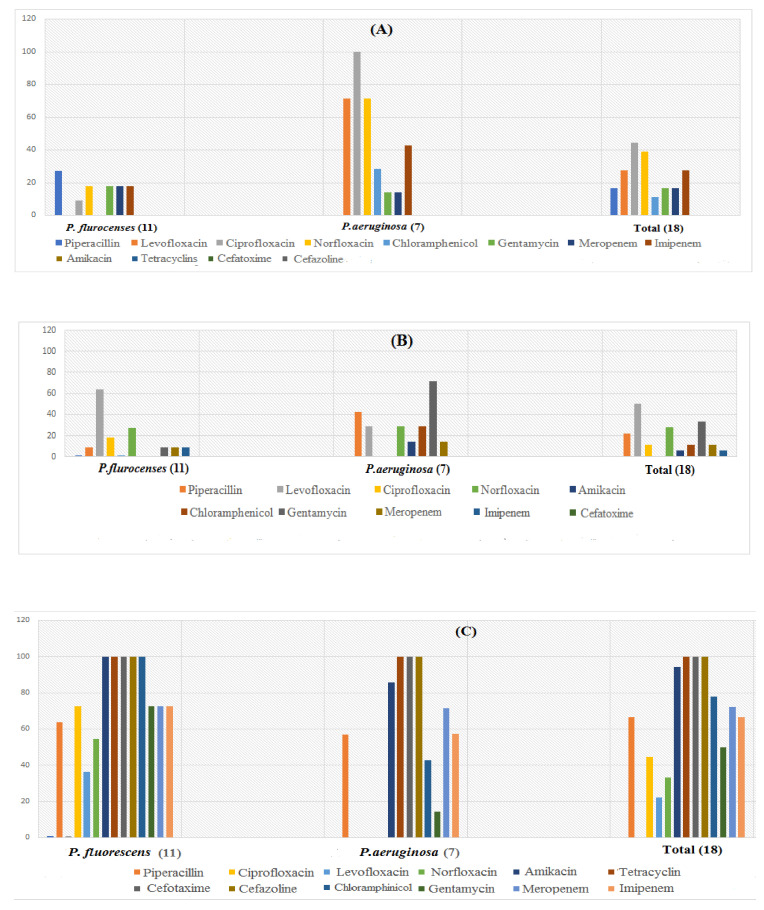
Results of an antimicrobial susceptibility test on *Pseudomonas* spp. isolates showed that they were variously sensitive (**A**), intermediate (**B**), resistant (**C**) to several antimicrobials.

After serological identification of eighteen *Pseudomonas* spp. isolates, of which seven were *P. aeruginosa* and eleven were *P. fluorescens*, these isolates were tested for biofilm formation and antimicrobial susceptibility. Antimicrobial susceptibility test results revealed that several *Pseudomonas* isolates had susceptibility to ciprofloxacin (44.4%) as noted in Figure 1A. Intermediate resistance was shown to levofloxacin (50%) followed by norfloxacin (27.8%) Figure 1B, while high resistance was shown against tetracycline, cefotaxime, and cefazoline (100%), followed by amikacin (94.4%), chloramphenicol (77.87%), meropenem (72.22%), imipenem (66.67%) and piperacillin (66.6%). It was noted that all *Pseudomonas* isolates were resistant to the β-lactam group as shown in Figure 1C.

Of eighteen *Pseudomonas* isolates, the results of phenotypic screening for *AmpC* β-lactamases revealed that 70% (13/18) of them were cefotaxime resistant. Two different confirmatory procedures, the boronic acid and disc tests, were used to identify isolates that produced *AmpC* β-lactamase. Fifteen of the eighteen isolates (83.3%) had positive results in both tests.

**Figure 2:**
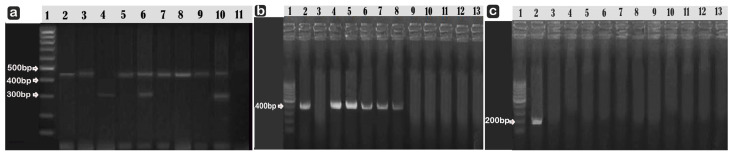
(**a**) Multiplex PCR products of the amplified *bla_CMY_* and *bla_MIR_* in *Pseudomonas* spp. were electrophoresed on agarose gel (lanes 2–5 *P. aeruginosa* and lanes 6–10 *P*. *fluorescens*) Lane 1 was a ladder with 100 base pairs; lanes 2, 3, 5, 6, 7, 8, 9, and 10 were positive isolates for *bla_CYM_* at 462. Lanes 4, 6, and 10 are *bla_MIR_* at 302 bp positive. lane 11 was a negative control. (**b**,**c**) Using uniplex PCR and agrose gel electrophoresis, *DHA* was amplified to a 405 bp length (**b**), Lane 1:M: 100 bp, Lanes 2: Positive Control, Lanes 4, 5, 6, 7, and 8: Positive Isolates. For the *FOX* gene (**c**), none of our *Pseudomonas spp* isolates displayed amplification for this gene at 190 bp, Lane 1:M: 100 bp, Lane 2, the positive control.

The PCR results for *AmpC* β-lactamases genes (*bla_CMY_*_-type genes_, *bla_MIR_*_-type genes_, *DHA* and *FOX*) confirmed the amplification of *bla_CYM_*_-type genes_ in all *Pseudomonas* species isolates (100%) while only three (27.2%) isolates of *P. fluorescens* possessed *bla_MIR_*-type genes, while no *P. aeruginosa* isolates showed amplification of *bla_MIR_*_-type genes_ as shown in Figure 2a. The *DHA* gene was detected in 42.8% (3/7) of *P. aeruginosa* and in 18.2% (2/11) of *P. fluorescens* isolates (Figure 2b). The *FOX* gene was not amplified in any of our isolates (Figure 2c).

Because most of our *Pseudomonas* isolates exhibited high resistance to the β-lactam group and extended-spectrum cephalosporins in addition to having β-lactam resistant genes, these isolates were classified as ESBL-*AmpC* combinations (extended-spectrum β-lactamases (ESBL) combined with plasmid-mediated *AmpC*-lactamases).

**Figure 3:**
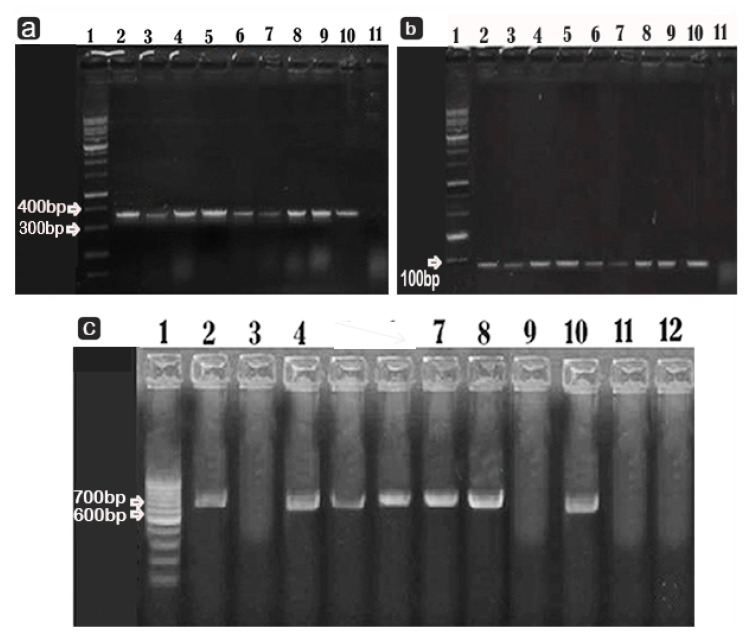
PCR results for biofilm formation genes (**a**–**c**) in *Pseudomonas* spp were detected on agarose gel electrophoresis. 369 bp for *pslD* (**a**), 89 bp for *rhlA* (**b**), and 786 bp for *pelA* are the expected molecular sizes of amplified DNA (**c**). lane 1 is DNA ladder, lanes 3–10 (3–6 *P. aeruginosa* and lanes 7–10 *P. fluorescens*) are isolates, lane 2 is positive control, and lane 11 is negative control.

In the microtiter plate results, seven *P. aeruginosa* strains produced biofilm (four strongly and three moderately), 8 *P. fluorescens* strains produced biofilm (four strongly and 4 moderately), and three strains were negative for biofilm on the microtiter plate, as shown in Table 2. Additionally, the results of the biofilm gene analysis showed that six (85.7%) of *P. aeruginosa* isolates and nine (81.8%) of *P. fluorescens* isolates carried *pslD* Figure 3a, while 5 (71.4%) of *P. aeruginosa* isolates and eight (72.7%) of *P. fluorescens* isolates carried *rhlA* Figure 3b. (Figure 3c). displays the *pelA* gene in two *P. fluorescens* isolates (18.1%) and four *P. aeruginosa* isolates (57.1%).

Table 3 shows that both *Pseudomonas* species were multidrug-resistant isolates for more than three classes of antimicrobials, particularly the *Pseudomonas* isolates resistant to β-lactam antibiotics, which showed the ability to form a strong and moderate biofilm.

**Figure 4:**
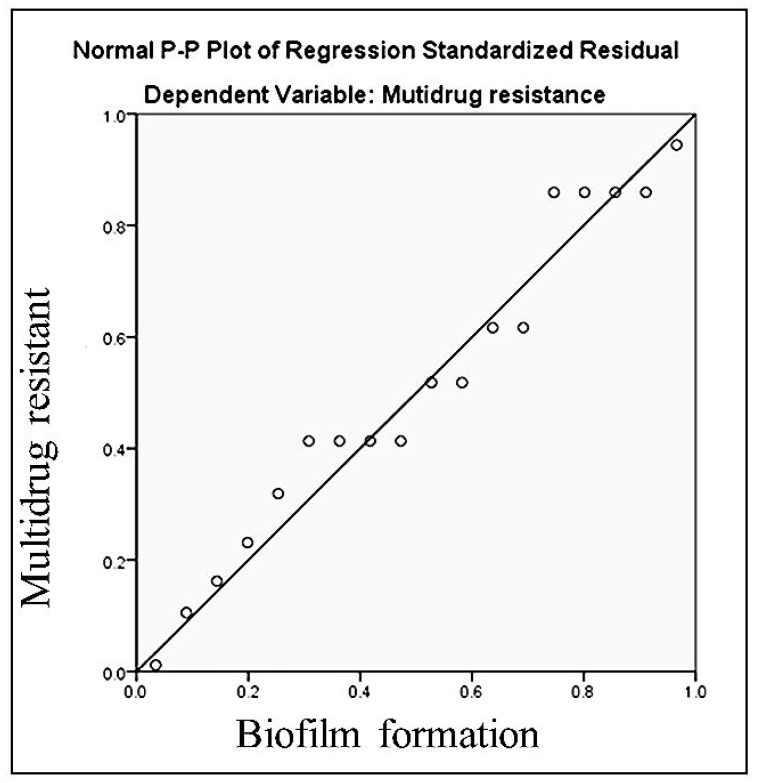
The relation between multidrug resistance and biofilm formation in *Pseudomonas* spp.

Figure 4 illustrates the statistically correlated significant association between MDR and biofilm formation isolates (*p* ˂ 0.05 and *r* = 0.6). In *Pseudomonas* isolates with varying percentages for antibiotics resistance, PCR confirmed the dispersion of β-lactamase resistance genes (*bla_CYM,_ bla_MIR_, DHA*, and *FOX*) and biofilm formation genes (*rhlA, pelA,* and *pslD*) (Table 4).

**Figure 5:**
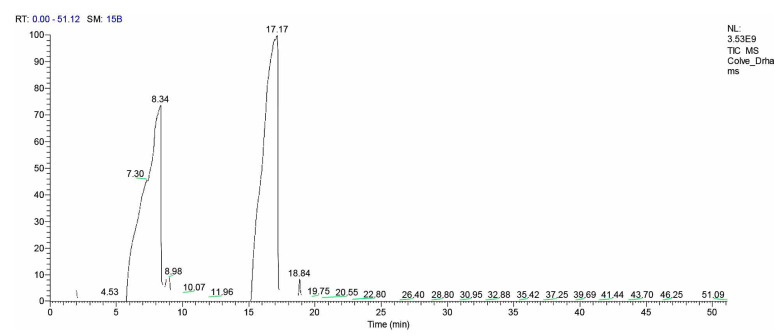
Gas chromatography–mass spectrometry (GC-MS) analysis of clove oil.

Using GC-MS Figure 5, the components of the clove oil were analyzed. Table 5 lists the active substances along with the retention time, molecular formula, molecular weight, and relative concentration (%) for the various constituents.

**Figure 6:**
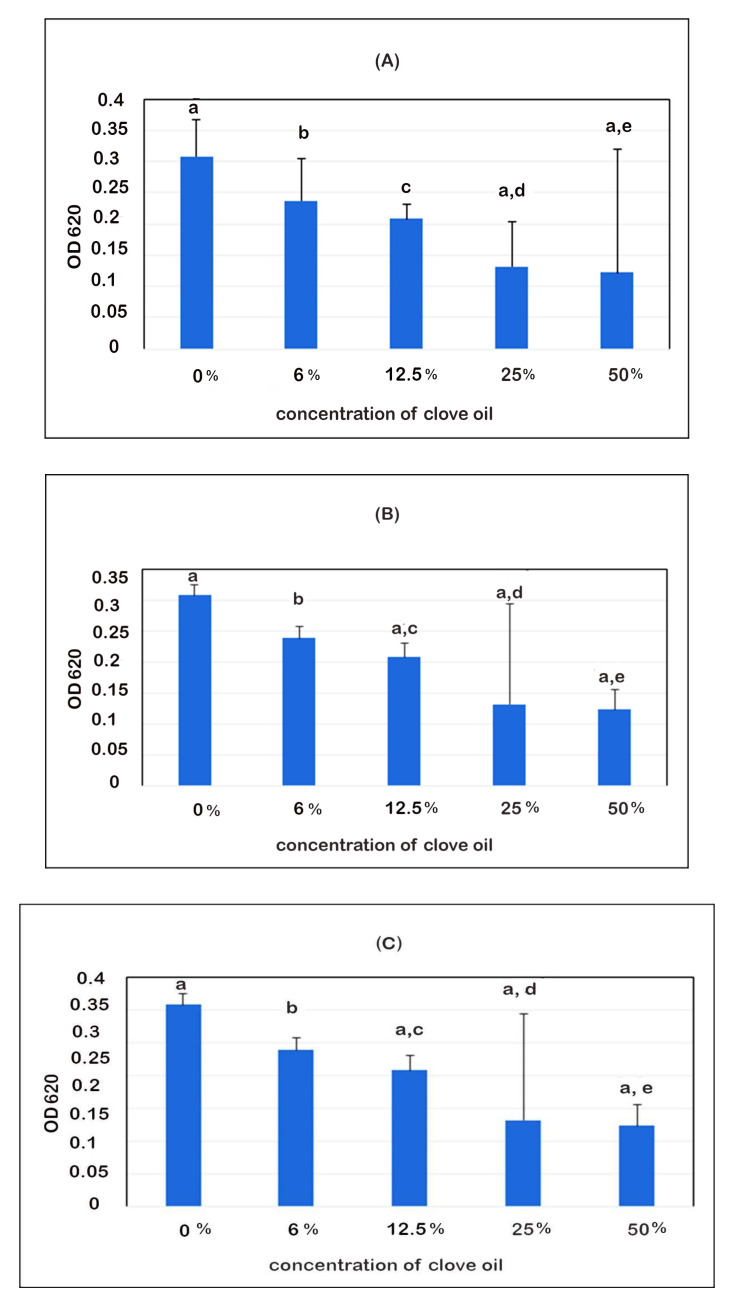
Quantitative analysis demonstrating how clove oils affect the growth of *Pseudomonas* spp. biofilms. *Pseudomonas* spp. biofilm development was measured in the presence of clove oil following 24 h culture in 96-well plates (620). (**A**) Clove oil’s antibiofilm properties against strong biofilms *P. aeruginosa* isolates; (**B**,**C**) clove oil’s antibiofilm properties against moderate biofilms *P. aeruginosa* isolates.

In this study, the ability of clove oil to inhibit *Pseudomonas* biofilm formation was investigated. The findings showed that biofilm production decreased with increasing clove oil concentrations with significant value (*p* ˂ 0.05, *r* = 0.67), and clove oil’s activity against *Pseudomonas* biofilm”. It was noticed that clove oil showed an antibiofilm effect at MIC ≥ 25% in strong biofilm formation isolates of *Pseudomonas* spp. (Figure 6A) and at MIC ≥ 12.5% concentrations in moderate biofilm formation isolates (Figure 6B,C) on the microtiter plate at (OD620).

## 4. Discussion

*P. aeruginosa* is one of the six bacterial species that make up the acronym ESKAPE, which often belong to multidrug resistant species. *P. aeruginosa* is a key contributor to persistent infections because of its propensity to form biofilms, which are bacterial colonies that are hard for antibiotics to eliminate because they are enclosed in self-produced extracellular matrix. [46].

Most *Pseudomonas* isolates in this study were resistant to β-lactam antibiotics as well as expanded spectrum cephalosporins, with a resistance factor (100%). According to Blair et al. [47]’s clarifications of our findings, *P. aeruginosa* has a high level of intrinsic resistance to most antibiotics because of restricted outer membrane permeability, efflux systems that push antibiotics out of the cell, and production of antibiotic-inactivating enzymes such as β-lactamases.

In this study, several *Pseudomonas* isolates demonstrated sensitivity to ciprofloxacin while displaying high resistance to different antibiotics (Figure 1). This finding supported by many authors with respect to of *Pseudomonas* especially *P. aeruginosa* showing resistant to different classes of antibiotics [48,49,50]. According to Hosu et al. [48] and Mohanty et al. [49], planktonic isolates of *Pseudomonas* are naturally resistant to larger antibiotic molecules because the bacterial cell membrane is impermeable. A few types of antibiotics can cross the outer membrane via a porin channel, which explains why certain *Pseudomonas* spp. are sensitive to them. 

In this study, several phenotypic plasmid-mediated *AmpC*-lactamases tests were conducted, including screening for *AmpC* β-lactamases, which provided an initial indication regarding the production of lactamases, and confirmatory tests such as the boronic test and the *AmpC* β-lactamase test which demonstrated that Tris-EDTA increases the release of β-lactamases by increasing the permeability of Gram-negative cells, and its inhibition of carbapenem activity improved the specificity of the test by avoiding cefotixin hydrolysis by these enzymes. The results of *AmpC* β-lactamases screening showed that 72.2% of *pseudomonas* isolates were *AmpC* β-lactamases producers while the confirmatory test showed that 83.3% of *pseudomonas* isolates were positive for these tests [50].

According to several authors [51], clinical laboratories do not routinely perform phenotypic tests because the available phenotypic tests are inopportune, lacking in sensitivity and specificity, or require reagents that are difficult to obtain and are linked to potentially fatal false susceptibility errors in routine susceptibility tests [37,52]. For this reason, we planned to use PCR to verify these results. To find *AmpC* in various Gram-negative species, Pérez-Pérez and Hanson [53] used PCR analysis. They found six groups of plasmid-mediated *AmpC*, including *ACC, DHA, CMY, EBC, FOX,* and *MOX.* According to Helmy and Wasfi [54], *DHA* was followed by the *CIT* gene family as the most common gene found in the *AmpC* gene family. Here *bla_CMY_* was the most prevalent β-lactamase gene belonging to the *CIT* group among our *Pseudomonas* isolates (100%), followed by *bla_MIR_* (27.2%), as shown in our PCR screening for plasmid-mediated *AmpC* (*CMY, DHA*, and *FOX*) of *Pseudomonas* spp., shown in Figure 2.

Different *Enterobacteriaceae* species contained *bla_CYM_*, according to Wendorf et al. [55]. Haldorsen et al. [56], who demonstrated *bla_CMY_* was the most prevalent in Gram-negative bacteria and had been observed worldwide, provided support for the earlier findings. It is exciting to note that *bla_CMY_* was present in a variety of plasmid backgrounds, indicating that it might be mobilized as a smaller transferable fragment.

The widespread distribution of the *bla_CMY_* gene among *Enterobacteriaceae* may be traced to a particular transposon-like element called ISEcp1.The *DHA* gene was found in 27.7% of our isolates. Comparable results (23.5%) were obtained in Cairo, Egypt by Fam et al. [57], while Hosny and Kashif [58] discovered that 40% of isolates contained this gene. On the other hand, our isolates did not contain *FOX*. While Wassef et al. [39] discovered that *FOX* showed the highest prevalence rate, Helmy and Wasfi [54] only found the *FOX* gene in a small number of isolates (1.3%).

The positive results of the β-lactamases screening tests in certain *Pseudomonas* isolates were not differentiated by the phenotypic test, however the presence of *bla_CMY_* in all isolates points to the upregulation of *AmpC* β-lactamases genes that are carried on plasmids. Additionally, the fact that these genes can be detected by PCR while being inefficiently expressed phenotypically may help to explain false-negative results [50].

Most of our *Pseudomonas* isolates were identified as extended-spectrum β-lactamases (ESBL) combined with plasmid-mediated *AmpC*-lactamases (ESBL-*Amp* Combinations), because these isolates showed a high resistance to the β-lactam group, extende-spectrum cephalosporins and other types of antibiotics and it possessed plasmid-mediated *AmpC* (*bla_CMY_* and *bla_MIR_*) [58,59]. These results were the same for both antibiotic sensitivity testing and PCR. Both molecular and phenotypic analyses are required for these pathogens, which have been identified as complex-resistant microbes [60].

The most significant clinical concern is multidrug-resistant Gram-negative bacteria, particularly those with *AmpC* resistant plasmids. These bacteria spread through nosocomial infections and long-term care facilities. It is necessary to identify the genes involved in order to control the movement of this resistance mechanism and thereby limit the spread of these bacteria. In order to distinguish *AmpC*-mediated resistance from other β-lactamase resistance mechanisms, clinical laboratories are becoming more interested in using molecular identification techniques [61].

Biofilm production, especially in hospital settings, contributes to the spread of nosocomial infections, and is considered as the primary factor in the antibiotic resistance of *P. aeruginosa* isolates. In addition, biofilm serves as a defense mechanism against antibiotics and the immune system [62]. More than 83% of the isolates in this study were able to create biofilm on microtiter plates (Table 2) Numerous authors have studied *Pseudomonas* biofilm [63,64] and linked their capacity to produce biofilm to primary production of exopolysaccharide (EPS), which serves a variety of purposes, such as preserving microcolonies and enhancing resistance to various disinfectants and environmental stresses [65,66]. Bacteria grown in biofilm are much more resistant to antibiotics and other chemotherapeutics than bacteria grown in planktonic form [67].

The etiology of our isolates’ virulence, that is, that they possess the biofilm formation genes *PslD* and *PelA,* was confirmed by PCR results. The *PslD* gene plays a crucial role in the beginning of biofilm formation and in protection of biofilm structure [17], while the *rhlA* gene control the next stage of biofilm maturation and development [42]. The *pel* gene encodes for exopolysaccharide that is sensitive to cellulose and contains 1–4 linked, partially acetylated galactosamine and glucosamine sugars [68]. The prevalence of *PslD* was 100% in our study, as shown in Figure 4, which was high compared to the findings of Banar et al. [69], who found *PslD* in 54.65% and *rhlA in 77.7% of* clinical isolates from an Iranian hospital.The *rhlA* was present in 77.7%, whiel Karami et al. [70] found that *rhlA* was present in all isolates, particularly MDR isolates. The different prevalent clones in each region, the species studied, and the length of the study all had an impact on the prevalence and type of acquired genes that Empel et al. [68] were able to identify. The pel (pellicle) operon regulates the development of a layer of polymer and cells at the air–liquid boundary of a *P. aeruginosa* standing culture, which is known as pellicle formation [71,72]. Hou et al. [43] and Ghadaksaz et al. [73] found that 31.03% and 45.2% of their isolates possessed *PelA*, respectively. In our study, *PelA* was detected in 33.3% of the isolates.

*Pseudomonas* isolates’ biofilms cause a loss in antimicrobial sensitivity, and infections caused by such isolates require the use of higher antibiotic concentrations [69]. Additionally, previous authors have demonstrated that the susceptibility of biofilms to antibiotics changes over time. Seven biofilm-producing *P. aeruginosa* isolates were found to be sensitive to certain members of the antibiotic family such as fluoroquinolone in earlier studies [74]. Over time, biofilm-producing *P. aeruginosa* strains isolated from pork, beef, and poultry developed resistance to enrofloxacin [75].

Our findings revealed a significant relationship between MDR and biofilm production (*p* < 0.05 and r = 0.67) (Figure 5). When compared to sensitive bacteria, MDR isolates were found to have a significantly higher propensity to form biofilms, according to research by Abidi et al. [76] and Corehtash et al. [77] Concerns about the link between biofilms and antibiotic resistance have been raised by biomedical researchers. This might be due to the role that biofilms plays as a model reservoir for cellular exchange of resistance-encoding plasmids, which encourages antibiotic resistance and promotes the horizontal transfer of genes that confer resistance [77]. Several authors have supported the relationship between antibiotic resistance and biofilm formation *P. aeruginosa* isolates [78,79,80].

Because EOs made from plants have a wide range of terpenoid and phenolic contents, they are frequently used as antibacterial and flavoring agents [26]. Many EOs have undergone extensive research into their antibacterial and antifungal properties [81]. EOs have been researched in other developmental contexts such as biofilm formation inhibition, toxin production inhibition, and bacterial quorum-sensing inhibition because traditional antimicrobial agents, which are primarily designed to inhibit cell growth, frequently result in bacterial drug resistance.

Caryophyllene, eugenol, and eugenyl acetate are just a few of the various components that make up clove oil. According to the GC-MS results (Figure 5), Eugenol is main bioactive component of clove essential oil. Chaieb et al. [24] and Heredia-Guerrero et al. [25] stated that eugenol is antimicrobial because of its capacity to irreversibly increase the permeability of cell membranes and destroy plasmatic membrane integrity [82]. Additionally, it is known to form antibiofilms at various concentrations [82,83], and is regarded as a safe compound [28].

In our study, clove oil has high antibiofilm activity of 85% against *Pseudomonas* species (Figure 6). This finding is supported by Zhou et al. [27], who claimed that eugenol and its derivatives are essential antibiofilm agents against *Pseudomonas aeruginosa.* Additionally, a number of EOs have antibacterial and antibiofilm effects against a range of pathogenic bacteria [27,81,84,85]. Eugenol has previously been discovered to prevent the development of biofilms by *Candida albicans* [85], *Staphylococcus aureus* [86], *Aspergillus flavus* [87], *Listeria monocytogenes* [88], and to inhibit quorum sensing of *P. aeruginosa* [27].

The risk of drug resistance can be decreased by overcoming the bacterial biofilm formation issue and creating biofilm-tolerant antimicrobials. The identification of the active ingredient and chemical structure activity relationship of eugenol and its derivatives reveals these substances’ antibiofilm activities. The mechanism of biofilm inhibition and virulence attenuation by eugenol and eugenol-rich oils against pathogenic bacteria has previously been revealed by transcriptional and phenotypic assays [83,89].

## 5. Conclusions

Most *Pseudomonas* isolates contain antimicrobial-tolerant biofilms. It is necessary to continually monitor the antimicrobial susceptibility of isolates that form biofilms in order to empirically solve empirical antimicrobial drug resistance issues. Due to their antibacterial and antibiofilm characteristics, EOs are a viable option. Many initiatives have been made to assess the potential application of EOs in the treatment of diseases caused by antibiotic resistant bacteria.

## Figures and Tables

**Table 1:** Primers used in this study.

Genes	Primer Sequence (5′–3′)	Amplicon Size (bp)	Annealing Temperature °C	References
* bla * *_ CMY _*	F-5′-TGG CCA GAA CTG ACA GGC AAA-3′	462	60	[38]
	R-5′-TTT CTC CTG AAC GTG GCT GGC-3′			
* bla * *_ MIR _*	F-5′-TCG GTA AAG CCG ATG TTG CGG-3′	302	60	
	R-5′-TTT CTC CTG AAC GTG GCT GCT GGC-3′			
*DHA*	F-5′-AAC TTT CAC AGG TGT GCT GGG T-3′	405	55	
	R-5′-CCG TAC GCA TAC TGG CTT TGC-3′			
*FOX*	F-5′-AAC ATG GGG TAT CAG GGA GAT G-3′	190	55	
	R-5′-CAA AGC GCG TAA CCG GAT TGG-3′			
*pslD*	F-5′-TGTACACCGTGCTCAACGAC-3′	369	58	[39]
	R-5′-CTTCCGGCCCGATCTTCATC-3′			
*rhlA*	F:5′-TGCTGATGGTTGCTGGCTTTC-3′	89	58	
	R:5′-CTCGGTGGTGATGGCATTCG-3′			
*pelA*	F:5′-CATACCTTCAGCCATCCGTTCTTC-3′	786	60	[40]
	R:5′-CGCATTCGCCGCACTCAG-3′			

**Table 2:** ELISA assay of biofilm formation among isolates of *Pseudomonas* spp.

Pseudomonas Isolates	No. (%) Strong Biofilm Producer Isolates	No. (%) Moderate Biofilm Producer Isolates	No. (%) Weak/Non Biofilm Producer Isolates
P. aeruginosa (n = 7)	4 (57.14%)	3 (42.86%)	-
P.fluorescens (n = 11)	4 (36. 36%)	4 (36.36%)	3 (27.27%)
Total (18)	8 (44.4%)	7 (38.8%)	3 (16.6%)

Reference strain. *P. aeruginosa* ATCC 27853, Mean OD at 620, STD 0.854716667 ± 0.063921496.

**Table 3:** Antibiotic resistance and biofilm formation results of *Pseudomonas* isolates.

Antibiotic	Bacterial Strains	Biofilm Formation Positive	Biofilm Formation Negative	Total Resistance Strains (%)
Piperacillin	*P. aeruginosa*	4 (57.1%)	-------	4 (57.1)
	*P. fluorescens*	4 (36.3%)	3 (27.2%)	7 (63.6)
Levofloxacin	*P. aeruginosa*	-----	---	------
	*P. fluorescens*	3 (27.2%)	1 (9.1%)	4 (36.3)
Ciprofloxacin	*P. aeruginosa*	------	-----	-----
	*P. fluorescens*	7 (63.6%)	1 (9%)	8 (72.7)
Gentamicin	*P. aeruginosa*	1 (14.2%)	------	1(14.2)
	*P. fluorescens*	5 (45.4%)	3 (27.2%)	8 (72.7)
Cefotaxime	*P. aeruginosa*	7 (100%)	-------	7 (100)
	*P. fluorescens*	8 (72.7%)	3 (27.2)	11 (100)
Cefazoline	*P. aeruginosa*	7 (100%)	-----------	7 (100)
	*P. fluorescens*	8 (72.7%)	3 (27.2)	11 (100)
Imipenem	*P. aeruginosa*	4 (57.2%)	-------	4 (57.2)
	*P. fluorescens*	5 (45.5%)	3 (27.27%)	8 (72.7)
Meropenem	*P. aeruginosa*	5 (71.4%)	------	5 (71.4)
	*P. fluorescens*	6 (54.5.4%)	2 (18.1%)	8 (72.7)
Norfloxacin	*P. aeruginosa*	0 (0%)	---	2 (28.5)
	*P. fluorescens*	4 (36.3%)	2 (18.1%)	6 (54.5)
Amikacin	*P. aeruginosa*	6 (85.7%)	---	6 (85.7)
	*P. fluorescens*	8 (72.7%)	3 (27.27%)	11 (100)
Tetracyclines	*P. aeruginosa*	7 (100%)	-----	7 (100)
	*P. fluorescens*	8 (72.7%)	3 (27.2)	11 (100)
Chloramphenicol	*P. aeruginosa*	3 (42.8%)	---	3 (42.8)
	*P. fluorescens*	8 (72.72%)	3 (27.27%)	11 (100)

**Table 4:** PCR results of biofilm formation and antimicrobial resistance genes in *Pseudomonas* spp.

Gene	*P. aeruginosa* (No = 7)	*P. fluorescens* (No = 11)	Total (No. of Isolates = 18)
Biofilm genes			
*PslD*	6 (85.7%)	9 (81.8%)	15 (83.3%)
*pelA*	4 (57.1%)	2 (18.1%)	6 (40%)
*rhlA*	5 (71.4%))	9 (81.8%)	14 (77.8%)
*AmpC* β-Lactamases genes			
*bla*_CMY-type genes_	7 (100%)	11 (100%)	18 (100%)
*bla*_MIR-type genes_	----	3 (27.2%)	3 (16.6%)
*DHA*	3 (42.8%)	2 (18.1%)	5 (27.7%)
*FOX*	-	-	-

**Table 5:** The phytochemical composition of clove essential oil by GC-MS.

Peak	R.t *	Name	Area %	Molecular Weight	Molecular Formula	MF **
1	8.34	Benzyl alcohol	37.12	108	C7H8O	958
2	8.98	PHENOL, 2-METHYL	1.07	108	C7H8O	801
3	17.17	Eugenol	61.81	164	C10H12O2	958

* R.t: retention time of different compounds of clove oil; MF **: Matching factor between mass spectrum of each compound and Mass Spectra database.

## Data Availability

The data presented in this study are available upon request from Hams M.A. Mohamed and Waleed Younis.
